# Letter to the Editor

**DOI:** 10.36469/001c.74185

**Published:** 2023-04-12

**Authors:** Ishir Narayan

**Affiliations:** 1 Indus Valley World School, Kolkata, India

**Keywords:** catastrophic health expenditure, sub-Saharan Africa, cardiovascular diseases, health economics, finance

## Abstract

Highlighting other risk factors for cardiovascular disease could enhance Adeniji and Obembe’s study.

In their article, “Cardiovascular Disease and Its Implication for Higher Catastrophic Health Expenditures Among Households in Sub-Saharan Africa,” Adeniji and Obembe^1^ have provided an excellent overview of catastrophic health expenditures in sub-Saharan Africa. The authors highlight the impact of cardiovascular diseases (CVD) on catastrophic health expenditure (CHE), concluding that “CVD predisposed households to risk of higher CHE.”

The authors deserve congratulations for this excellent report. However, certain issues merit further discussion and need to be highlighted. First, while the authors have chosen tobacco use, alcohol use, and servings of fruits and vegetables, they have omitted other important risk factors like the prevalence of diabetes, hypertension, truncal obesity, and dyslipidemia, which could have been easily captured in the study and would have provided a more comprehensive risk factor assessment for CVD. The reason behind the extremely significant difference in tobacco use between Ghana and South Africa (6.6% vs 73.84%) among patients with CVD is inexplicable. While similar low smoking rates in Ghana have been reported previously,^2^ the South African smoking rate reported by the authors appears abnormally high and could represent a sampling bias. Previous publications have put the smoking rate in South Africa between 17.6% and 20.5%.^3,4^

The authors report that “households with CVD were more likely to experience greater CHE across all the thresholds examined in Ghana.” However, from the confidence intervals provided in **Table 6**, it appears that at the 25% threshold, CVD status was not a risk factor for CHE. Similarly, in South Africa, CVD status was not a risk factor for CHE at the 5%, 10%, and 40% thresholds. Further, insurance was not a risk factor in Ghana at any of the thresholds, while it was an important risk factor in South Africa at all thresholds except 10%. Perhaps the authors could have provided further insight into this variation.

Multiple definitions exist for calculating CHE and include not only several thresholds (10%-40%) for defining CHE,^5,6^ but also significant variations in the variables used within a given threshold.^6^ While the report is laudable for analyzing CHE at various thresholds, it also highlights the need to arrive at a single universally agreed definition for CHE to allow comparisons across different studies and geographic regions.

Ishir Narayan

Indus Valley World School, Kolkata, India
